# Female excellence in rock climbing likely has an evolutionary origin

**DOI:** 10.1016/j.crphys.2021.01.004

**Published:** 2021-02-06

**Authors:** Collin Carroll

**Affiliations:** Columbia University. 2 Broad Street, Westport, CT, 06880, USA

**Keywords:** Rock climbing, Human evolution, Arboreal locomotion, Performance gap, Sex-blind musculoskeletal adaptations

## Abstract

The human body is exceptional for many reasons, not the least of which is the wide variety of movements it is capable of executing. Because our species is able to execute so many discrete activities, researchers often disagree on which were the movements most essential to the evolution of our species. This paper continues a recently introduced analysis, that the performance gap between female and male athletes narrows in sports which most reflect the movements humans evolved to do. Here, I examine the performance gap in rock climbing. Female climbers are some of the best in the world irrespective of gender, a trend that is not found in any other major sport. I conclude that the exceptional ability of female rock climbers relative to male rock climbers is further evidence of the existence of sex-blind musculoskeletal adaptations, which developed over the course of human evolution – as a result of external (non-sexual) selection forces – to facilitate essential movements. These adaptations abate some of the general physical sexual dimorphism which exists in humans. This paper provides more evidence that the human body was shaped, in part, by pressure to climb well.

## Highlights

• Rock climbing is the sport most similar to tree climbing, a movement essential to human development.
• Multiple women can be found in the list of top 100 rock climbers, a trend not found in any other major sport.
• Sports with a higher degree of gender equity, may reflect movements with a greater degree of evolutionary importance.
• Rock climbing’s gender gap provides further evidence that early humans faced external selection pressure to climb well.
• Thus, the importance of climbing to the survival of humans - even after the onset of genus *Homo* - may be understated.

## Graphical abstract

**Figure:**
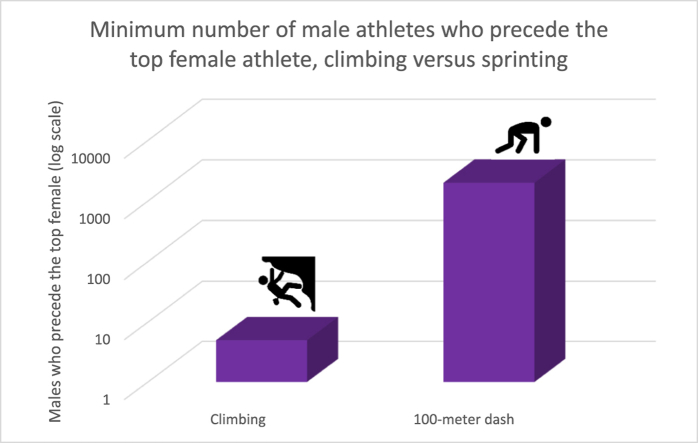

“It goes, boys!”

-Lynn Hill, after becoming the first person to free climb The Nose on El Capitan

## 1 Introduction

### 1.1 Background

While evolutionary anthropologists generally agree on much of the history of human development, there is still a great deal of debate as to which movements the human body has adapted. Most of this debate is specific to those movements that arose in the past few million years in conjunction with the genus *Homo*. Some anthropologists, for instance, claim that the ecological dominance of humans can be primarily attributed to our overhand throwing ability (Lombardo and Deaner 2018a; Roach et al. 2013; Wilson et al. 2016), another sect argues that endurance running has been critical to the development of our species (Bramble and Lieberman 2004; Liebenberg 2006; Lieberman et al. 2006), and others still make the case that humans adapted to excel at intraspecies hand-to-hand combat (Carrier 2011; Carrier et al. 2015). These are only a few perspectives on the evolutionary history of *Homo sapiens*, each supported by its own set of evidence.

It is generally accepted, however, that for the millions of years before the emergence of *Homo*, human ancestors were capable tree climbers (Green and Alemseged 2012; Stern and Susman, 1983, Wood and Baker, 2011). Even now, as is the case with other primates, humans remain capable climbers; a number of researchers have observed modern hunter-gatherers climbing trees to acquire resources (Ichikawa 1981; Venkataraman et al. 2012). Arboreal locomotion does appear to be an essential part of our development as a species.

The importance of climbing is imprinted in the human musculoskeletal system: our long arms, short trunk, and upright posture all appear to have originated to facilitate arboreal locomotion (Crompton et al. 2008; Fleagle 2013; Thorpe et al. 2007). These physiological features are seen in both the male and female members of our species, and with that in mind, this paper aims to analyze the climbing ability in humans using a recently introduced metric: the performance gap (PG) between males and females in sport. A recent paper (Carroll 2019) showed that sports which show a smaller difference in ability between male and female athletes may be most similar to movements essential to the evolution of *Homo sapiens*. A narrower gender PG in rock climbing ability would suggest that humans were deeply shaped by the selection forces that advantaged good climbers.

This paper examines the PG between males and females in rock climbing, the sport most similar to the arboreal movement that was essential to the origination of our species, to identify whether climbing does indeed have a narrow PG. If true, this would provide more evidence that the forces of natural selection made our ancestors better climbers, and it suggests that climbing may have had a more important role in the development and subsequent survival of our species than currently thought.

### 1.2 Evolutionary evidence of arboreal movement to the development of modern humans

There is considerable evidence supporting the idea that arboreal movement was crucial to the survival of early humans. Before the emergence of the genus *Homo*, human ancestors relied on a lifestyle that seems to be a mix of arboreal and terrestrial in nature to avoid predators and access resources (Senut 2014; Stern 2000). And while tree-climbing behavior in early humans almost certainly preceded bipedalism, there is debate as to whether bipedalism first descended from upright posture in the trees or whether it emerged from knuckle-walking patterns of terrestrial locomotion (Begun 2004; Gebo 1996; Prost 1980; Schmitt 2003; Senut et al. 2018; Stern and Susman 1981). Regardless of the exact transition from arboreal to terrestrial locomotion in humans, it is clear our ape ancestors led a life in the trees.

While the exact degree to which *Australopithecus afarensis,* for instance, moved and lived arboreally is in dispute, it seems clear that the species spent a significant amount of time in the trees and held adaptations needed for considerable arboreal locomotion (Crompton et al. 2008; Crompton et al., 2011; Duncan et al. 1994; Green and Alemseged 2012; Stern 2000). The fossil record of the australopith A.L. 288-1 (“Lucy”), as one example, shows evidence of substantial reliance on climbing behavior (Jungers 1982; Kappelman et al., 2016; Meyer et al. 2015; Ruff et al. 2016).

The importance of tree climbing is not limited to our distant ancestors, however; evidence of arboreal locomotion can be seen in the genus *Homo*, too. Antón and Snodgrass (2012), comparing *Homo* to *Australopithecus*, found that both genera have a locomotor repertoire that is significantly dependent on arboreal movement. Indeed, early hominins do evince a number of features that can be linked to frequent arboreal behavior (Roberts et al. 2016; Susman et al. 1984; Tuttle 1981). One of these features, for instance, is a robust upper limb that suggests a high tolerance for mechanical loading, which would have been necessary for a life spent at least partially in trees (Haeusler and McHenry 2007; Heinrich et al. 1993; Ruff 2009; Susman and Creel 1979). Features like this provide evidence of the importance of arboreal locomotion to early *Homo*.

Beyond the importance it had to early *Homo*, arboreal locomotion appears to be significant to our own species. Of course, *Homo sapiens* is better suited to bipedal terrestrial locomotion than to arboreal locomotion, given how energetically economical walking is compared to climbing in humans (Elton et al. 1998; Kozma et al., 2018). But even with this clear preference, humans do retain many traits that facilitate tree climbing. Compared to smaller, primarily arboreal primates, humans do not expend significantly more energy climbing per kilogram of body mass, meaning we are still efficient climbers (Hanna et al. 2008). The human hand, with its “power” grip designed to facilitate prehensile movements as described by Napier (1956), also allows for arboreal locomotion; additionally, humans have been measured using subtle proprioceptive measures, like “light touch” fingertip support, to reduce bipedal instability and improve balance in tree-canopy-like environments (Johannsen et al., 2017). Even the gluteus maximus muscle, essential for bipedal locomotion, is particularly active during climbing, suggesting that some adaptations which favor terrestrial bipedalism also have use in arboreal movement (Bartlett et al. 2013). The human mind also appears to have arboreal origins as well. Povinelli and Cant (1995) suggest the idea of self-conception – the cognitively advanced ability that allows an organism to perceive of itself – in large great apes and humans originally developed to better facilitate arboreal clambering. And an experiment of human tree climbers by Hanson (2016) found that most made conscious choices to better facilitate their movement up a tree. Perhaps the most compelling evidence of human tree climbing, though, comes from behavioral studies of select modern hunter-gatherer populations, who frequently climb trees as a method of resource acquisition (Kraft et al. 2014). In short, while the exact degree to which tree climbing has been useful to *Homo sapiens* is yet undetermined, plenty of evidence suggests that humans remain well adapted to arboreal locomotion.

### 1.3 Rock climbing as a modern analog to tree climbing

As a result of millions of years of arboreal locomotion, the ability to climb is ingrained in the human form. This ability is not limited to climbing trees exclusively; certain physiological and cognitive traits found in humans also facilitate rock climbing. One is the strength of the human hand. Grip and finger strength are unsurprisingly crucial in rock climbing; elite rock climbers possess stronger grips and better finger and hand endurance than less experienced rock climbers and non-climbers (Cutts and Bollen 1993; Ozimek et al., 2017; Philippe et al. 2011). Additionally, the position of the human body during climbing further improves grip strength. According to Parvatikar and Mukkannavar (2009), grip strength is highest when the shoulder is positioned in 180° of flexion, the exact positioning of a climber grasping onto a hold above his or her body. Our muscular endurance is another key to our species' rock-climbing ability; humans appear to have a greater relative VO_2_max – a measurement of an organism's maximal oxygen consumption – than other apes (Pontzer 2017). Booth et al. (1999) suggest that “outdoor rock climbing might require a large fraction of the climber's peak oxygen uptake,” so our high VO_2_max advantages climbing in humans. The human mind is crucial to our rock-climbing ability, too. Rock climbers, when compared to untrained controls, have improved visual-spatial perception (Marczak et al., 2018), and expert rock climbers also have better visual and motor memory when compared to climbers with less expertise (Whitaker et al. 2019). Because rock climbing and tree climbing are similar, and because competition tree climbing is effectively nonexistent, rock climbing serves as the athletic analog of tree climbing in this paper. This paper expands on the idea that the PG across sports shrinks or narrows depending on how relevant a sport is to human evolution; climbing sports – given the considerable evidence of the role climbing has played in human development – would be expected to have a relatively narrow PG.

## 2 Materials and methods

### 2.1 How climbing ability is assessed

Unlike many single-competitor sports such as track & field or Olympic lifting, which are standardized to allow for easy comparison between competitors, ability in rock climbing is measured by the competitors, who “rate” the climbs they complete based on difficulty. In this manner, the athletes in rock climbing act as the judges of the sport as well as its record keepers. Once a rock climber completes a particular route for the first time, he or she is responsible for its rating so that other climbers will know the difficulty of the route. These athletes grade rock climbing routes according to different scales; for the sake of clarity, the Yosemite Decimal System (YDS) is used in this paper unless otherwise noted.

The YDS is divided into 5 main classes, with Class 5 used to grade rock climbing routes. Class 5 is divided into categories, with 5.15 being the most difficult that currently exists. Further subcategories a-d exist within each route categorized as 5.10 and above. Thus “Silence,” the most difficult rock-climbing route ever climbed, holds a rating of 5.15d (Skenazy 2017). Climbing ability tends to be measured as a climber's “best ascent” – that is, the most challenging route a climber has climbed – to measure his or her skill. Only those elite climbers who have climbed a 5.15-rated route are noted here.

### 2.2 Climbing data

This paper uses data aggregated by rock climber and journalist William Kuelthau (Kuelthau, 2020). This list was last updated on February 4th, 2020, and the data gathered and analyzed on March 24, 2020. The climbers who have climbed a 5.15-grade route, 90 in all, are included in this data set by best ascent so that no climber is recorded more than once. The data here are measured conservatively, so any climb specifically marked as questionable by Kuelthau will not be included in the set.

### 2.3 Limitations of climbing data

The data on outdoor rock climbing suffers chiefly from a lack of standardization, bifurcated into two main issues: first, rock climbing records are not held by any dedicated organization; second, there is some subjectivity in the way routes are rated by climbers.

Rock climbing as a sport lacks an official record keeper responsible for tracking which athletes have climbed which routes. Organizations like the International Federation of Sport Climbing exist, but their purpose is more to establish rock climbing competitions on artificial courses than to keep track of outdoor free climbing and bouldering ascents. By contrast, track & field is governed by an official body, World Athletics, which maintains records for all official track & field events for both men and women (“World Athletics”, 2020). The lack of an official body governing outdoor rock climbing means the data on ascents is of inherently lower quality than that of the data on track & field events. Because climbing results are self-reported rather than recorded at official events, unscrupulous climbers have more freedom than track & field athletes to make dishonest claims about whether they have actually completed the routes which they have climbed. However, this is unlikely to significantly affect climbing results at the highest level, 5.15 and above, because the attempts of these climbers are typically witnessed by others and the results often captured on video, providing a record of the ascents (“Gripped”, 2017). As stated earlier, any climbs marked as questionable are not included in my data set.

Another issue with rock climbing records is that they are measured qualitatively, by the climbers who are the first to complete each route. Track performance, on the other hand, is measured quantitatively, by an athlete's time to run a certain distance, for instance. The subjectivity of rock-climbing records, while problematic, is mitigated to an extent. Experienced rock climbers do indeed tend to rate routes accurately, according to a study by Draper et al. (2011). If the original climber gives a route an inappropriate rating – one that is too easy or too difficult for the route – it can be changed based on the collective input of subsequent climbers (Pesterfield 2018). This dynamism does mitigate some of the subjectivity of the rating system, especially at the upper reaches of the sport, where rating difficult routes can become a collaborative effort between the few climbers capable of ascending the world's toughest climbs (Lucas 2017). Because ascending a route rated 5.15a or higher is such a rarity in climbing, the added scrutiny makes it more difficult for a climber to inflate an easier route to a grade in the 5.15 range. Fewer than 100 climbers have ever climbed a 5.15-rated route, and the most accomplished of these elite climbers – those who have climbed a 5.15b or higher, for instance – can confirm or deny 5.15 status for many of these extremely difficult climbs (“Gripped”, 2018). For the purposes of this paper, it can be inferred that the ratings of the most difficult outdoor rock-climbing routes in the world – 5.15a through 5.15d – tend to accurately reflect the actual difficulty of these routes; any climber who has climbed a 5.15-rated route is very likely to be one of the top rock climbers of all time.

## 3 Theory

### 3.1 The performance gap in sport as a measure of human evolution

In recent years, the PG on the whole has stabilized, meaning the current difference between male and female athletes at any given sport is a good indicator for the difference between overall male and female ability – the idea being that elite athletes reflect the upper limits of human capability (Millard-Stafford et al. 2018). A previous paper published on the subject of the PG as a tool for measuring evolutionary movements dealt exclusively with track & field events; the extremely high-quality data from that study strongly suggested that there was a significantly narrower PG in short-distance sprinting events when compared to longer-distance events, and that there was also a significantly narrower PG in running events of all distances compared to jumping events (Carroll 2019). Because it was evolutionary advantageous for humans, regardless of gender, to sprint well – to escape predators, primarily – the paper argued that forces of natural selection led to an accrual of traits that made for better sprinters in both males and females. These traits are termed sex-blind musculoskeletal adaptations (SBMA's). SBMA's bridge the general gap of physical dimorphism that exists between men and women (a result of sexual selection), which in turn leads to a narrower PG in the sports most similar to movements humans adapted to do.

The sport of rock climbing, analogous to arboreal locomotion, would be expected to have a particularly narrow PG if early humans and human ancestors faced considerable external (non-sexual) selection that in turn honed their abilities as tree climbers. The purpose of this paper is to explore whether or not this hypothesis holds true. If it does, this paper would provide further evidence that the human body accrued a number of SBMA's in service of climbing, which could help clarify the evolutionary history of *Homo sapiens*. Such evidence could prove a jumping-off point to further investigate the relative importance of climbing, and to analyze other movements using the PG.

## 4 Results

**Fig. 1:**
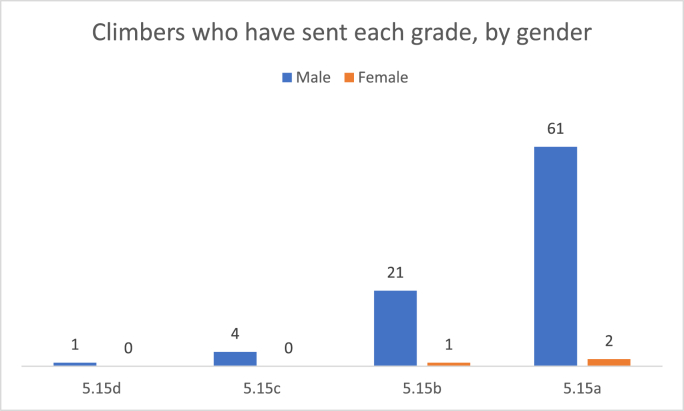
Number of climbers, by gender, who have climbed each of the four routes graded most difficult.

**Fig. 2:**
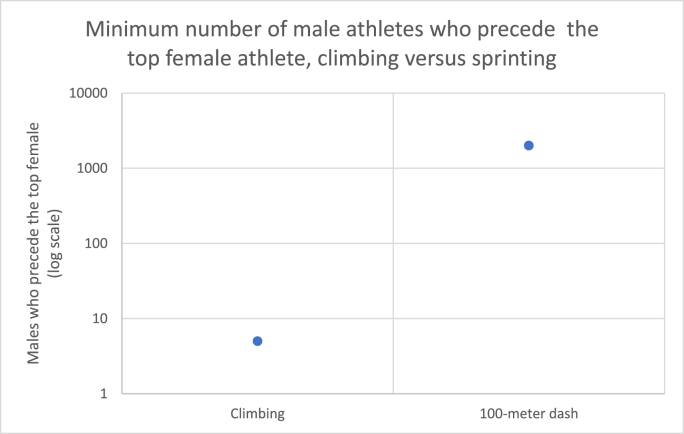
Minimum number of male athletes who have outperformed the top all-time female athlete in climbing versus running. A much greater number of male runners are likely to precede the top female in the 100-m dash than even shown here, but records online are limited to approximately 2000 sprinters per gender.

Table 1 and Fig. 1 summarize the number of climbers, by gender, who have climbed each of the four most difficult ratings, 5.15d through 5.15a. Supplementary data of each of the climbers by name can be found in Appendix 1. With one female climber who has climbed a 5.15b-rated route and two others who have climbed 5.15a-rated routes, females compose 3 of the top 90 climbers of all time. Fig. 2 shows how many male athletes, at minimum, have eclipsed the top female athlete in rock climbing versus how many male sprinters are quicker than the fastest female at the 100-m dash. Data on the 100-m dash come from WorldAthletics.com. This figure aims to create a sense of scale to better express how close in ability the top female rock climber is to the top male climbers when compared to how close the top female sprinter is to the top male sprinters. Because the data listed by World Athletics is cut off at a certain time – 10.30 s for the 100-m dash– they do not represent the total number of male sprinters who have eclipsed the top female sprinter; it is likely that many thousands more male sprinters are faster than the top female.

**Table 1:** Number of climbers, by gender, who have climbed each of the four routes graded most difficult. Climbers are listed once, by their best ascent.

	5.15d	5.15c	5.15b	5.15a	Total
**Male**	1	4	21	61	87
**Female**	0	0	1	2	3

## 5 Discussion

### 5.1 Female ability in rock climbing relative to other sports

As hypothesized, relative female rock-climbing ability was shown to be extraordinary. A female climber has climbed a 5.15b-rated route, one of only 27 people to have successfully ascended a route rated 5.15b or higher. Additionally, this exceptional performance is not limited to one female outlier. Two other women have climbed 5.15a-rated routes, placing 3 females in the top 90 climbers of all time. This level of female achievement is far beyond that seen in other sports. The 100-m dash, with a relatively narrow PG itself, does not have a single female runner in the top 2,000 competitors, and the fastest female time ever recorded is slower by 0.19 s than the 2000th-fastest-ever male time. This trend holds true for the marathon, too. The top female does not enter into the top 2,000 marathon runners, and she is more than 2 min slower than the 2,000^th^-fastest male (“World Athletics” 2020). This means that there are likely many thousands more male runners who surpass the world-record-holding females in each track & field event. Rock climbing as a sport shows a much narrower PG at its upper echelon than either short-distance or long-distance running.

A sport like rock climbing, with its numerous exceptional female athletes, exhibits a remarkably narrow PG. This is likely as a result of the duration of time early humans spent climbing trees as a means of survival. Arboreal locomotion was essential to our development as a species, and its importance has led to the development of many climbing specific SBMA's. The idea behind SBMA's is that certain traits – like a high strength-to-weight ratio, in the case of tree climbing – have been particularly selected for in early humans. These traits bridge the gap of general sexual dimorphism that exists between men and women. Males, due to their body composition and size, hold a general advantage over females in physical activity, but the presence of a greater number of climbing specific SBMA's shrinks this gap and therefore the PG in climbing sports. Capable tree climbers, male and female, could avoid predators and access resources unavailable to others, allowing them to survive and reproduce. That the PG in climbing is so narrow suggests the forces of natural selection favored capable climbers throughout much of the history of *Homo sapiens* and its ancestors.

### 5.2 Limitations

This analysis of the PG has its limitations. As stated earlier, the data on climbing is not standardized, nor was it able to be gathered from a dedicated rock-climbing organization. Additionally, because the track data offered by World Athletics is cut off at certain times for men, I was unable to accurately count the number of men who out-achieved the overall top woman in the 100-m dash. Still, the data does appear to accurately reflect how relatively narrow the PG is between the top men and women in rock climbing relative to other sports. Even though the quality of the data may be low, it is still clear that, when compared to other physical activities, women are exceptional rock climbers.

Another limitation is the difficulty in comparing rock climbing to track & field. Rock climbing is qualitative, whereas track & field is quantitative; thus, the PG two sports cannot be evaluated in the same manner. To bridge this gap, the total number of males who exceed the top female in each sport was used in Fig. 2. This comparison, however, is confounded by a difference in total participation sizes in each sport; running practitioners greatly outnumber rock climbers – by a factor of approximately six, according to Statista (Lange, 2020, 2021b), a market and consumer research firm – which could explain in part the number of men who outperform the top woman in track and field. Were current rock-climbing participants to be scaled up to those of running (increased sixfold), or arbitrarily increased tenfold, or fiftyfold, though, the result would still be the same: fewer male rock climbers would surpass the top female rock climber than would male sprinters who surpass the top female sprinter. Additionally, a total participation gap is unlikely to explain the sheer magnitude of the PG in running versus climbing: the top high school male in the state of Connecticut ran the 100-m dash in 10.48 s, whereas the top female high-school sprinter in Connecticut ran the event in 11.50 s (My Track & Field Records, 2020). The disparity here equates to a PG of 91.1 – using the metric laid out by Carroll (2019) – which is a level similar to that of the PG between all sprinters collectively. This means that even in smaller populations, the PG appears to be relatively stable. Rock climbing almost certainly holds a much narrower PG than does track & field regardless of participation rate.

### 5.3 Differing interpretations of the PG between men and women

This perspective is not the only one regarding the PG and human evolution. Some have argued that a wider PG should be used as the evolutionary metric, because it indicates a level of differential selection between the sexes – one that favors males – at certain movements. Morris et al. (2020), for example, found that men could produce much more relative force than women during a movement that simulated punching versus overhead pulling. And, also using the data set from World Athletics described in Carroll (2019), Lombardo and Deaner (2018b) argue that overhead throwing movements like the javelin show a wider PG than either running or jumping as a result of a differential selection favoring males who were superior throwers. These authors all claim that as a result of an evolutionary pressure favoring punching-capable or throwing-capable males, the PG would be wider in punching and throwing movements.

I disagree with these analyses. It is the norm for men to greatly outperform women in physical ability, not the exception. Many other sports exhibit similarly wide PG's to throwing: for the heaviest classes of the Olympic lift, the world-record-holding female currently lifts 68.6% the weight of world-record-holding male (“IWF” 2020a; “IWF” 2020b). This PG is lower than the PG of 73.4% between female and male javelin throwers, although it should be noted that this analysis is confounded by weight difference in throwing sports; women throw a 600g javelin, whereas males throw one that is 800g, and by the fact that these Olympic lifting records only reflect two years of competition due to a restructuring of weight classes by the sport's governing body. Still, were a wider PG linked to differential selection that favored better male throwers, we would not expect such a wide gap between male and female lifters. This example, while certainly limited, does provide more evidence that a wide PG between males and females is the norm and that a narrow PG, like that of rock climbing's PG, is more noteworthy. While narrow PG's can help determine which movements were essential to overcoming external selection forces in our species, more research may be needed to determine if wider PG's develop as a result of sexual selection.

Another interpretation of the PG to address here is the potential impact of the participation disparity between male and female athletes: if a greater proportion of rock climbers are female compared to the proportion of track & field athletes who are female, this argument goes, the PG will be artificially narrowed. Similarly, if a greater proportion of rock climbers are male than in other sports, the PG in rock climbing would be expected to artificially widen. This reasoning may be valid, but in practice, the participation disparity does not appear to be correlated with the PG. It is addressed by Carroll (2019), who found that a greater proportion of female high-school runners participate in cross-country (an analog of long-distance running) than outdoor track & field (an analog of sprinting). Even with this participation disparity, the PG in high-school long-distance running was comparable to that of all-time adult long-distance running, and the PG in high-school sprinting was comparable to that of all-time adult sprinting. Thus, the PG appears to be relatively unaffected by subtle differences in the participation disparity. If anything, the participation disparity argument would provide *more* evidence speaking to the narrowness of rock climbing's PG. The ratio of rock climbers by gender, according to an estimate by professional climber Sasha DiGiulian, is 60% men to 40% women (Dwyer 2019), a wider gender participation disparity than, for instance, the one found in high-school outdoor track & field, which in 2019 was approximately 55% men to 45% women (“National Federation of State High School Associations” 2019). A subtle participation gap by sex is unlikely to alone explain the extraordinary ability of top female climbers and thus the narrow PG in rock climbing.

### 5.4 Expected advantages female climbers have over males

A number of traits likely developed to better facilitate arboreal locomotion; these traits are ones that could close the PG between male and female rock climbers. Firstly, men have a higher bone mass than women (Nieves et al., 2005), which adds additional weight that a man must overcome to ascend a rock face. A high strength-to-weight ratio is crucial in climbing, and additional bone mass would add more weight to males, decreasing that ratio. Secondly, it has been well documented that women on average are more flexible than men, with a greater degree of joint mobility doing movement (Ferber et al. 2003; Kato et al., 2005; Mendiguchia et al. 2011; Rene 1984). Rock climbing is likely better enabled by increased flexibility, and more-capable rock climbers have been found to possess greater flexibility metrics than less-capable ones (Draper et al. 2009; Grant et al. 1996). It should be noted that some studies, however, have called into question the degree to which flexibility impacts climbing performance (Mermier et al. 2000; Wall et al. 2004). Finally, and perhaps most importantly, women have been shown to possess greater endurance than men; females are able to maintain isometric contraction for a significantly longer period of time over the same level of relative intensity (Clark et al. 2003; Hicks et al. 2001; Hunter et al. 2004). This could be in part because women generally have a greater proportion of slower-contractile skeletal muscle fibers, fibers which facilitate endurance rather than power output (Haizlip et al. 2015; Welle et al. 2008). Females, though possessing a lower amount of grip strength, have been shown to have levels of forearm and hand endurance equal to or greater than, those of males (Dipla 2017; Fulco et al., 1999; Gonzales and Scheuermann 2007; Hunter 2014). Females may also be more resistant to fatigue than men as a result of their better muscular activation strategies (Nie et al., 2007). This makes for a climbing advantage in females, muscular endurance being a key component in rock climbing and time to exhaustion a key indicator of a climber's ability (España-Romero et al., 2009; Mackenzie et al., 2020; Mermier et al., 2000; Giles et al. 2006; Sheel 2004). These traits – lower bone mass, greater flexibility, and better relative endurance – are three physiological examples that could explain in part the extraordinary ability seen in the top female rock climbers.

### 5.5 Expected advantages male climbers have over females

Interestingly, other traits that make for better climbers are more pronounced in men. A low body fat percentage, for one, is particularly important in climbing (Giles et al. 2006; Watts et al. 1993). The body-fat percentage of women is typically higher than that of men, even in elite athletes and rock climbers specifically (Fleck 1983; Mitchell et al. 2011; Novoa-Vignau et al. 2017). Men, in addition to having greater muscle mass as a percentage of their bodies than women, hold a greater relative percentage of their muscle mass in their upper body versus their lower body (Janssen et al. 2000). Men also have a cognitive advantage over females in climbing, their heightened visual-spatial processing power (Weiss et al. 2003). Spatial perception and mental rotation tasks, both of which climbers would rely on during an ascent, are easier for men than for women (Linn and Petersen, 1985; Pietsch and Jansen 2018). Taken all together, there do appear to be a number of physiological and cognitive advantages men in general would have over women in climbing, yet women are still adept climbers. This all suggests that the climbing-specific SBMA's and sex-blind cognitive adaptations present in humans are numerous and substantial enough to mitigate many of the expected advantages men would have over women in climbing. It also provides evidence that the PG itself may be a better indicator of the evolutionary importance of a movement in our species than individual measures like muscle fiber composition or spatial reasoning.

### 5.6 How SBMA's bridge the gender gap in climbing

A number of attributes are required for successful rock climbing; many of them have been discussed so far in this paper. Some traits, like better flexibility and muscular endurance, are more pronounced in women. Others, like increased upper body muscle mass and heightened visual-spatial reasoning, are more pronounced in men. A third category of traits likely exists, traits that facilitate climbing for both women and for men, and into this category fall the SBMA's that close the PG between men and women. The structure of the human body – with its many large back muscles, strong hands, and mobile joints – enables tree climbing in a number of ways. The makeup of the pulley system in the hand is one example, as it may have developed to create friction while climbing as a mechanism of fall prevention (Schöffl et al., 2009). These specific adaptations which advantage climbing in humans also overcome the general advantage men have at physical activity, a process which manifests itself as a narrower PG between the sexes in the sport of rock climbing.

SBMA's have arisen in response to natural evolutionary stressors human ancestors faced, like the threat of terrestrial predators, which could explain the narrower PG found in sprinting versus endurance running, for example (Carroll 2019). The longer and more important a movement like climbing, or sprinting, was to facing external selection forces in early humans, the narrower the PG in corresponding movements should be. A narrow PG does not necessarily indicate recency, though: many of the physiological and psychological traits which facilitate rock climbing in modern humans likely first developed in earlier hominid (and pre-hominid) ancestors. The narrow PG in rock climbing, however, suggests that the human body is still well-equipped to climb, which could indicate that climbing trees for resource acquisition and for predator evasion may have been crucial for recent hominids and early *Homo sapiens* alike.

A thought experiment regarding the white-tailed deer may elucidate how the PG measures selection. The white-tailed deer faces a number of external selection factors, the inescapable presence of predators chief amongst them. To this end, the whitetail has evolved tremendous speed as an evasive ability. The whitetail also must deal with intra-species selection: males rut for access to females. Bucks, therefore, are advantaged by characteristics that make for good rutters (most obviously, their antlers). The rutting “PG” between male and female white-tailed deer would be – clearly – very wide. No research could be found, however, claiming that bucks are better sprinters than does. Even though bucks are noted to be approximately one-third larger, according to SUNY ESF's Adirondack Ecological Center (2020), they do not seem to have an advantage sprinting. Evading predators (dealing with external selection forces) is required for deer of either sex, so the sprinting “PG” in deer should be expected to be narrower than the rutting “PG,” which does appear to be the case. In deer, forces associated with external selection seem to narrow the PG, and forces associated with sexual selection seem to widen it.

The PG in sport can help explain which movements the human body is best equipped to execute in a way that other metrics cannot. The different types of running discussed by Carroll (2019) exemplify this. Despite bearing features like a smaller proportion of “fast-twitch” muscle fibers and a smaller stride length, females are better sprinters than endurance runners relative to men (Haizlip et al. 2015; Welle et al. 2008). Females *should* be expected, given their physiology, to be relatively better endurance runners than sprinters … but they are not. Short-distance sprinting was likely crucial to both our male and female ancestors as a means of predator evasion, so SBMA's that advantaged better sprinters gradually developed in humans, leading to the relatively narrow PG in sprinting today. Simply put, to overcome external selection forces, humans needed to be better sprinters than endurance runners.

As a result of the extraordinary ability of female climbers, rock climbing possesses a uniquely narrow PG. Although it cannot be ruled out that this may in fact be due in part to sex-specific traits found in females, the narrowness of rock climbing's PG – especially when compared to another sport with a relatively narrow PG, sprinting – suggests that climbing was a key movement to the survival and development of our species's ancestors. Whether due mostly to SBMA's or to sex-specific traits, though, the relative ability of top female climbers is unparalleled. Thus, the importance of climbing to the habits of early humans, and potentially to the habits of more recent hunter-gatherers, may be overlooked and should be explored more.

## 6 Conclusion

Arboreal locomotion is well documented as an integral part of human evolution, especially as it relates to primate evolution. A tremendous volume of evidence suggests that precursors to the genus *Homo*, early *Homo*, and even *Homo sapiens* have all climbed trees; a number of physical and psychological traits found in humans seem to have originated at least in part to facilitate tree climbing. Many of these traits can be considered SBMA's, ones common to both men and women, that neutralize the general physical differences between males and females. As a result of SBMA's bridging the physical gender gap, the PG between men and women narrows in sports that most closely mimic movements essential to human evolution. Rock climbing, an analog of tree climbing, exhibits a uniquely narrow PG between the sport's top female and male athletes. The extraordinary ability of female climbers, and the corresponding PG, provides additional evidence that climbing was a movement essential to human evolution.

## Credit author statement

William Kuelthau: Data Curation

Special thanks to the reviewers of this paper, for their diverse set of opinions in the review & editing of this paper

## Declaration of competing interest

The authors declare that they have no known competing financial interests or personal relationships that could have appeared to influence the work reported in this paper.

## Appendix A Supplementary data

Application Application
